# Supplementary material for: Inferring Atlantic salmon post-smolt migration patterns using genetic assignment
Source: R Soc Open Sci. 2019 Oct 2;6(10):190426. doi: 10.1098/rsos.190426 (PMC6837218; doi:10.1098/rsos.190426)
Supplement: Supplementary File 1 [file rsos190426supp1.docx]

**Supplementary File 1** **:Inferring Atlantic salmon post-smolt migration patterns using genetic assignment**

**Harvey A. C., Quintela M., Glover K. A., Karlsen, Ø., Nilsen, R., Skaala, Ø., Sægrov, H., Kålås, S., Knutar, S., Wennevik, V.**

**Methods**

*Population genetic summary statistics*

The total number of alleles and allelic richness of each river and samples of the post-smolts were calculated with Microsatellite Analyser (MSA) (Dieringer and Schlötterer, 2003). Pairwise F_ST_ and its significance after 10 000 permutations were tested among baseline rivers using ARLEQUIN v.3.5.1.2 (Excoffier et al., 2005). Pairwise F_ST_ and its significance were also calculated between temporal samples more than 3 years apart within baseline rivers.

*Population genetic structure*

To investigate potential assignment units based on hierarchical population genetic structure, two different clustering methods were used. STRUCTURE v.2.3.4 (Pritchard et al., 2000) was used to identify possible genetic groups among rivers under a model assuming admixture and correlated allele frequencies using the ParallelSTRUCTURE (Besnier and Glover, 2013) package in R. Ten runs with a burn-in period of 500 000 replicates and a run length of 1 000 000 Markov Chain Monte Carlo (MCMC) iterations were performed for clusters ranging from 1-14. STRUCTURE was rerun using the same criteria with geographical outlier populations (Tysseelva and Oselva, as these rivers are located in another fjord system) removed for clusters ranging from 1-12. STRUCTURE Harvester was then used to calculate the Evanno et al. (2005) ad hoc summary statistic ΔK, based on the rate of change of the estimated likelihood between successive K values, allowing the determination of the uppermost hierarchical level of structure in the data. Runs were averaged with CLUMPP v.1.1.1 using the LargeK-Greedy algorithm and the G’ pairwise matrix similarity statistic, and graphically displayed using bar plots. Secondly, StructureSelector (Li and Liu, 2017) was used to estimate four alternative statistics (MedMed, MedMean, MaxMed and MaxMean), which have been described as more accurate than the previously used methods to determine the real number of clusters, for both even and uneven sampling data. Then an analysis of principal component (PCA) approach was implemented in Genodive (Meirmans and Van Tienderen, 2004), using an eigen analysis on the covariance matrix between the allele frequencies of the baseline populations.

**Tables & Figures**

Table S1: Microsatellite primer information for the 31 markers. PCR Program refers to the amplification conditions which are described in Table S2.

| **Primers** | **Dye** | **Direction  (Forward/Reverse)** | **Sequences** | **References** | **PCR Program** |
| --- | --- | --- | --- | --- | --- |
|  |  |  |  |  |  |
| SSsp2210 | 6FAM | F | AAG TAT TCA TGC ACA CAC ATT CAC TGC | Paterson et al. 2004 | 1 |
|  |  | R | CAA GAC CCT TTT TCC AAT GGG ATT C |  |  |
| SSspG7 | PET | F | CTT GGT CCC GTT CTT ACG ACA ACC | Patterson et al. 2004 | 1 |
|  |  | R | TGC ACG CTG CTT GGT CCT TG |  |  |
| SsaD144 | NED | F | TTG TGA AGG GGC TGA CTA AC | King et.al 2005 | 1 |
|  |  | R | TCA ATT GTT GGG TGC ACA TAG |  |  |
| Ssa202 | 6FAM | F | CTT GGA ATA TCT AGA ATA TGG C | O'Reilly et al. 1996 | 1 |
|  |  | R | GTT CAT GTG TTA ATG TTG CGT G |  |  |
| Sp2201 | PET | F | TTA GAT GGT GGG ATA CTG GGA GGC | Patersson et al. 2004 | 1 |
|  |  | R | CGG GAG CCC CAT AAC CCT ACT AAT AAC |  |  |
| SsaD157 | NED | F | ATC GAA ATG GAA CTT TTG AAT G | King et.al 2005 | 1 |
|  |  | R | GCT TAG GGC TGA GAG AGG AAT AC |  |  |
| Ssa289 | PET | F | CTT TAC AAA TAG ACA GAC T | McConnell et al. 1995 | 2 |
|  |  | R | GTC ATA CAG TCA CTA TCA TC |  |  |
| Ssa14 | NED | F | CCT TTT GAC AGA TTT AGG ATT TC | McConnell et al. 1995 | 2 |
|  |  | R | CAA ACC AAA CAT ACC TAA AGC C |  |  |
| Ssa171 | NED | F | TTA TTA TCC AAA GGG GTC AAA A | O'Reilly et al. 1996 | 2 |
|  |  | R | GAG GTC GCT GGG GTT TAC TAT |  |  |
| Sp2216 | 6FAM | F | GGC CCA GAC AGA TAA ACA AAC ACG C | Paterson et al. 2004 | 2 |
|  |  | R | GCC AAC AGC AGC ATC TAC ACC CAG |  |  |
| Sp1605 | PET | F | CGT AAT GGA AGT CAG TGG ACT GG | Paterson et al. 2004 | 2 |
|  |  | R | CTG ATT TAG CTT TTT AGT GCC CAA TGC |  |  |
| SSsp3016 | NED | F | GAC AGG GCT AAG TCA GGT CA | *Genbank no. AY372820* | 3 |
|  |  | R | GAT TCT TAT ATA CTC TTA TCC CCA T |  |  |
| SsaF43 | 6-FAM | F | AGC GGC ATA ACG TGC TGT GT | Sanchez et al. 1996 | 3 |
|  |  | R | GAG TCA CTC AAA GTG AGG CC |  |  |
| SSa197 | PET | F | TGG CAG GGA TTT GAC ATA AC | O'Reilly et al. 1996 | 3 |
|  |  | R | GGG TTG AGT AGG GAG GCT TG |  |  |
| SsaD486 | NED | F | TCG CTG TGT ATC AGT ATT TTG G | King et.al 2005 | 3 |
|  |  | R | ACT CGG ATA ACA CTC ACA GGT C |  |  |
| MHC1 | VIC | F | AGG AAG GTG CTG AAG AGG AAC | Grimholt et al. 2002 | 3 |
|  |  | R | CAA TTA CCA CAA GCC CGC TC |  |  |
| MHC2 | VIC | F | GAT GGC AAA GAG GAA AGT GAG | Stet et al. 2002 | 3 |
|  |  | R | TTG TTA TGC TCT ACC TCT GAA |  |  |
| SsOSL85 | 6-FAM | F | TGT GGA TTT TTG TAT TAT GTT A | Slettan et. al. 1995 | 3 |
|  |  | R | ATA CAT TTC CTC CTC ATT CAG T |  |  |
| EST28 | 6FAM | F | CACAGGCACACACTCCTCAT | Vasemägi et al. 2005 | 4 |
|  |  | R | GTTTCAGGTGAAGAGCATGACCAA |  |  |
| EST19 KA-2R | VIC | F | CGCTTCCTGGACAAAAATTA | Vasemägi et al. 2005 | 4 |
|  |  | R | GTTTCATCTCTGTCATTCTCTTGC |  |  |
| Ssa407 | PET | F | TCGTGACTACTAAGTCTTTGACCA | Cairney et al. 2000 | 4 |
|  |  | R | GTTTGTGTAGGCAGGTGTGGAC |  |  |
| SSleer15-1 | 6FAM | F | CATGTGCGTGTGCTTTTACAG | GenBank U86708 | 4 |
|  |  | R | GTTTTCTGCATGTAGAACCCTGACC |  |  |
| SLEEN 82 | 6FAM | F | CATGGAGAATCCCACTTTCTTA | GenBank U86706 | 4 |
|  |  | R | GTTTCAGGGAGTGATATGGGACATAA |  |  |
| Sleel53 | 6FAM | F | TGATTTGTTGCCTGCTGCTTCC | GenBank U86704 | 4 |
|  |  | R | GTTTCCTGCTGCCCACATCATCC |  |  |
| Ssa412 | VIC | F | GTGGAGATACACAGCACTTA | Cairney et al. 2000 | 5 |
|  |  | R | GTTTCTTGGTTAGTACCGGACATG |  |  |
| Ssa405 | NED | F | CTGAGTGGGAATGGACCAGACA | Cairney et al. 2000 | 5 |
|  |  | R | GTTTACTCGGGAGGCCCAGACTTGAT |  |  |
| Ssa98 | NED | F | GCAGTCCTTACCTGTGTGATTA | O'Reilly et al. 1996 | 5 |
|  |  | R | GTTTGGTAGTGATCTGGAGAGTGC |  |  |
| Ssosl25 | NED | F | ATCTACACAGCTCCTGGTGGCAG | Slettan et al. 1995 | 5 |
|  |  | R | GTTTCATGTAATGGGTCGAGAGAAGTG |  |  |
| SSsp2215 | VIC | F | GGTCAGTCAGTCACACCATGC | Paterson et al. 2004 | 5 |
|  |  | R | GTTTAGGTGTCCTGCCGGTCAAT |  |  |
| EST107 | PET | F | AGCGTTACGTCGAATCCAA | Vasemägi et al. 2005 | 5 |
|  |  | R | GTTTCTCATGGAGGGTGGAAGTGT |  |  |
| EST68 | PET | F | TGACACTGTGGCCTGTCTCT | Vasemägi et al. 2005 | 5 |
|  |  | R | GTTTGAGTTCTGGGTTATTTATTCACA |  |  |

Table S2: PCR protocols for each microsatellite multiplex (1-5). All temperatures are in ⁰C and times are in seconds. For PCR program 5 use 30 cycles.

| PCR program 1: | |  |  |  |  |
| --- | --- | --- | --- | --- | --- |
|  |  |  |  |  |  |
|  |  | **Temperature (°C)** | | **Time** |  |
| Denaturation | | 94 | | 4 min |  |
| Denaturation | | 94 | | x 28 cycles   \| 50 s \| \| --- \| |  |
| Annealing | | 55 | | 50 s |  |
| Extension | | 72 | | 80 s |  |
| Extension | | 72 | | 10 min |  |
|  |  | 4 | | ¥ |  |
| PCR program 2: | |  |  |  |  |
|  |  |  |  |  |  |
|  |  | **Temperature (°C)** | | **Time** |  |
| Denaturation | | 94 | | x 26 cycles   \| 4 min \| \| --- \| |  |
| Denaturation | | 94 | | 50 s |  |
| Annealing | | 55 | | 50 s |  |
| Extension | | 72 | | 80 s |  |
| Extension | | 72 | | 10 min |  |
|  |  | 4 | | ¥ |  |
| PCR program 3: | |  |  |  |  |
|  |  |  |  |  |  |
|  |  | **Temperature (°C)** | | **Time** |  |
| Denaturation | | 94 | | 4 min |  |
| Denaturation | | 94 | | x 26 cycles   \| 50 s \| \| --- \| |  |
| Annealing | | 55 | | 50 s |  |
| Extension | | 72 | | 80 s |  |
| Extension | | 72 | | 10 min |  |
|  |  | 4 | | ¥ |  |
| PCR program 4 &5: | | |  |  |  |
|  |  |  |  |  |  |
| **Step** | **TEMP*** | **time (s)*** |  |  |  |
| 1 | 95 | 150 |  |  |  |
| 2 | 95 | 25 |  |  |  |
| 3 | 58 | 30 |  |  |  |
| 4 | 72 | 25 |  |  |  |
| 5 | GOTO step 2, 9 cycles |  |  |  |  |
| 6 | 95 | 25 |  |  |  |
| 7 | 53 | 30 |  |  |  |
| 8 | 72 | 25 |  |  |  |
| 9 | GOTO step 6, 28 cycles |  |  |  |  |
| 10 | 72 | 600 |  |  |  |
| 11 | 12 | 120 |  |  |  |
| 12 | 4 | ∞ |  |  |  |

Table S3: Pairwise F_ST_ (below diagonal) and P-values after 10000 permutations (above diagonal) for each river in the baseline. Note that Omvikelva (OM) was split into old (OM old: 2011, 2012) and new (OM new: 2016-2018) samples based on Fst results on these temporal samples.

|  | **OS** | **TY** | **ST** | **GR** | **EI** | **KI** | **OP** | **ÆN** | **RO** | **OM old** | **OM new** | **US** | **ET** | **ÅD** |
| --- | --- | --- | --- | --- | --- | --- | --- | --- | --- | --- | --- | --- | --- | --- |
| **OS** | - | 0.000 | 0.000 | 0.000 | 0.000 | 0.000 | 0.000 | 0.000 | 0.000 | 0.000 | 0.000 | 0.000 | 0.000 | 0.000 |
| **TY** | 0.019 | - | 0.000 | 0.000 | 0.000 | 0.000 | 0.000 | 0.000 | 0.000 | 0.000 | 0.000 | 0.000 | 0.000 | 0.000 |
| **ST** | 0.019 | 0.015 | - | 0.000 | 0.000 | 0.000 | 0.000 | 0.000 | 0.000 | 0.000 | 0.000 | 0.000 | 0.000 | 0.000 |
| **GR** | 0.018 | 0.016 | 0.003 | - | 0.000 | 0.000 | 0.000 | 0.009 | 0.000 | 0.000 | 0.000 | 0.000 | 0.000 | 0.000 |
| **EI** | 0.017 | 0.017 | 0.005 | 0.006 | - | 0.000 | 0.000 | 0.000 | 0.000 | 0.000 | 0.000 | 0.000 | 0.000 | 0.000 |
| **KI** | 0.017 | 0.016 | 0.004 | 0.004 | 0.005 | - | 0.000 | 0.000 | 0.000 | 0.000 | 0.000 | 0.000 | 0.000 | 0.000 |
| **OP** | 0.019 | 0.017 | 0.003 | 0.005 | 0.011 | 0.007 | - | 0.000 | 0.000 | 0.000 | 0.000 | 0.000 | 0.000 | 0.000 |
| **ÆN** | 0.014 | 0.014 | 0.005 | 0.006 | 0.013 | 0.008 | 0.007 | - | 0.000 | 0.000 | 0.000 | 0.000 | 0.018 | 0.000 |
| **RO** | 0.016 | 0.013 | 0.006 | 0.005 | 0.009 | 0.006 | 0.006 | 0.007 | - | 0.000 | 0.000 | 0.000 | 0.000 | 0.000 |
| **OM old** | 0.014 | 0.016 | 0.007 | 0.008 | 0.013 | 0.009 | 0.009 | 0.007 | 0.007 | - | 0.000 | 0.000 | 0.000 | 0.000 |
| **OM new** | 0.015 | 0.015 | 0.008 | 0.011 | 0.014 | 0.010 | 0.010 | 0.006 | 0.006 | 0.006 | - | 0.000 | 0.000 | 0.000 |
| **US** | 0.016 | 0.014 | 0.006 | 0.010 | 0.011 | 0.010 | 0.010 | 0.008 | 0.006 | 0.008 | 0.008 | - | 0.000 | 0.000 |
| **ET** | 0.007 | 0.013 | 0.008 | 0.006 | 0.011 | 0.007 | 0.008 | 0.004 | 0.004 | 0.007 | 0.008 | 0.007 | - | 0.000 |
| **ÅD** | 0.025 | 0.022 | 0.008 | 0.011 | 0.014 | 0.012 | 0.009 | 0.013 | 0.011 | 0.014 | 0.013 | 0.016 | 0.013 | - |

Table S4: Proportion of the baseline samples that were assigned to each river by the Leave One Out test in ONCOR. The diagonal (in bold) represents the proportion of individuals that were correctly self-assigned to their river of origin.

|  | **OS** | **TY** | **ST** | **GR** | **EI** | **KI** | **OP** | **ÆN** | **RO** | **OM** | **US** | **ET** | **ÅD** |
| --- | --- | --- | --- | --- | --- | --- | --- | --- | --- | --- | --- | --- | --- |
| **OS** | **0.52** | 0.04 | 0.00 | 0.00 | 0.00 | 0.04 | 0.00 | 0.04 | 0.00 | 0.11 | 0.07 | 0.19 | 0.00 |
| **TY** | 0.02 | **0.85** | 0.02 | 0.02 | 0.00 | 0.02 | 0.00 | 0.00 | 0.02 | 0.02 | 0.02 | 0.02 | 0.00 |
| **ST** | 0.00 | 0.07 | **0.44** | 0.07 | 0.15 | 0.04 | 0.11 | 0.00 | 0.04 | 0.04 | 0.04 | 0.00 | 0.00 |
| **GR** | 0.00 | 0.00 | 0.04 | **0.31** | 0.12 | 0.08 | 0.19 | 0.08 | 0.04 | 0.08 | 0.04 | 0.04 | 0.00 |
| **EI** | 0.00 | 0.00 | 0.10 | 0.10 | **0.59** | 0.06 | 0.02 | 0.00 | 0.00 | 0.04 | 0.04 | 0.02 | 0.02 |
| **KI** | 0.00 | 0.00 | 0.00 | 0.13 | 0.00 | **0.63** | 0.00 | 0.00 | 0.00 | 0.13 | 0.13 | 0.00 | 0.00 |
| **OP** | 0.00 | 0.00 | 0.07 | 0.04 | 0.07 | 0.06 | **0.54** | 0.01 | 0.03 | 0.03 | 0.04 | 0.09 | 0.01 |
| **ÆN** | 0.00 | 0.00 | 0.00 | 0.00 | 0.00 | 0.00 | 0.08 | **0.25** | 0.00 | 0.25 | 0.33 | 0.00 | 0.08 |
| **RO** | 0.00 | 0.02 | 0.02 | 0.05 | 0.00 | 0.02 | 0.02 | 0.02 | **0.41** | 0.05 | 0.20 | 0.20 | 0.00 |
| **OM** | 0.00 | 0.00 | 0.01 | 0.00 | 0.02 | 0.02 | 0.01 | 0.02 | 0.07 | **0.76** | 0.04 | 0.02 | 0.01 |
| **US** | 0.00 | 0.01 | 0.03 | 0.00 | 0.01 | 0.01 | 0.04 | 0.01 | 0.03 | 0.10 | **0.64** | 0.10 | 0.01 |
| **ET** | 0.03 | 0.01 | 0.02 | 0.03 | 0.02 | 0.02 | 0.02 | 0.02 | 0.05 | 0.06 | 0.08 | **0.63** | 0.01 |
| **ÅD** | 0.00 | 0.00 | 0.06 | 0.17 | 0.00 | 0.00 | 0.11 | 0.00 | 0.06 | 0.06 | 0.06 | 0.17 | **0.33** |

Figure S1: Outputs of the principle component analysis for (A) the full baseline and (B) the baseline without Oselva (OS) and Tysse (TY).


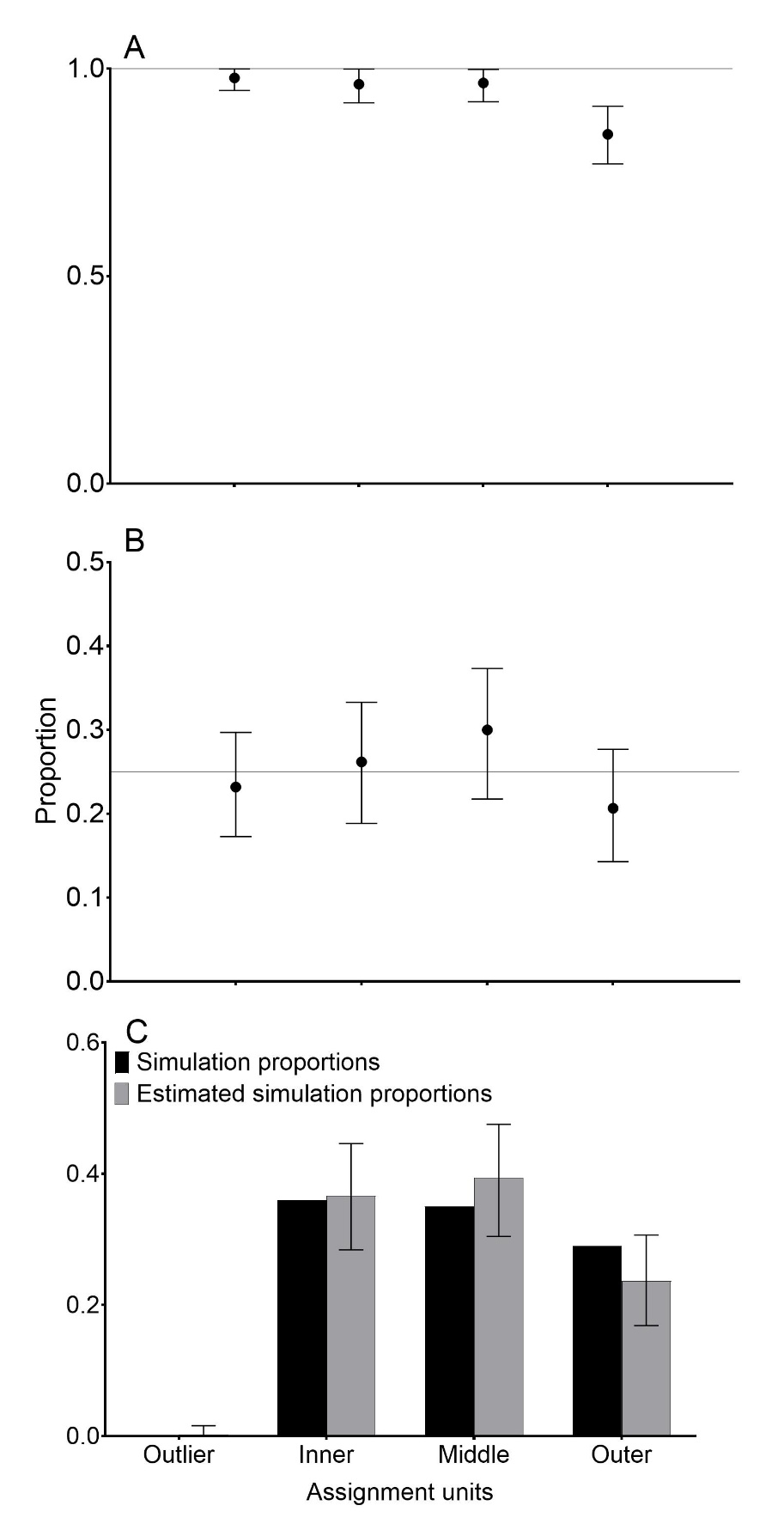


Figure S2: Results of the fishery simulations for the regional assignment units. A) 100% simulations, B) realistic fishery simulation with equal proportions for each assignment unit, C) realistic fishery simulation with the proportions of each assignment unit based on the estimated smolt production from reports from 2008. For A and B, the horizontal grey lines represent actual simulated proportions and the points represent the simulated mean proportions. For C, the black bars represent simulated proportions and the grey bars represent the mean estimated simulation proportions. For all plots the error bars represent the 95% confidence intervals.


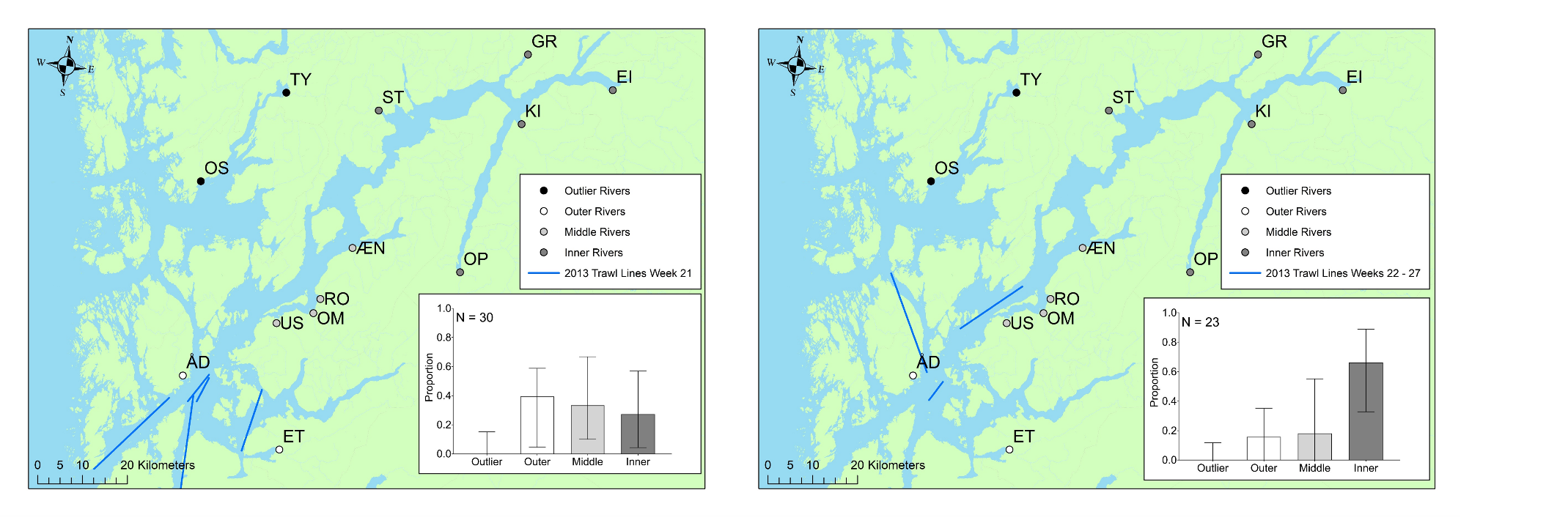


Figure S3: Weekly mixed stock analysis proportions of trawl fish caught in the 2013 trawl survey and trawl lines for each week. Note that weeks 22 -27 have been merged due to low numbers of fish caught during that time.


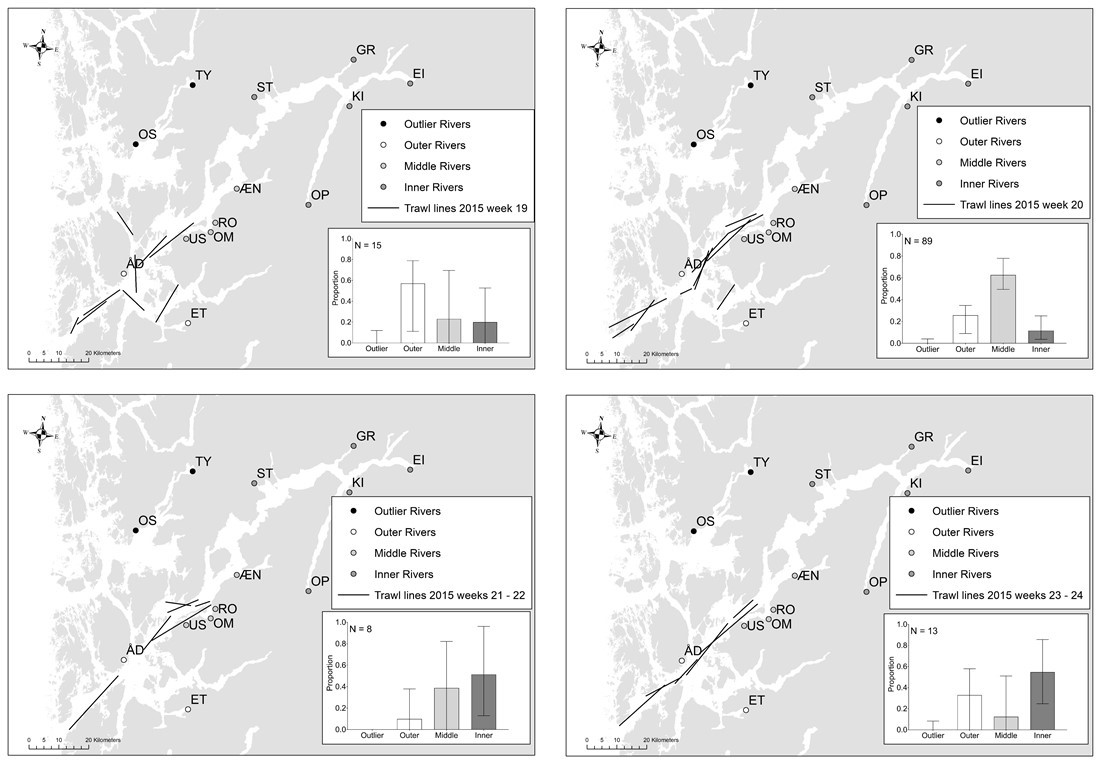


Figure S4: Weekly mixed stock analysis proportions of trawl fish caught in the 2015 trawl survey and trawl lines for each week.


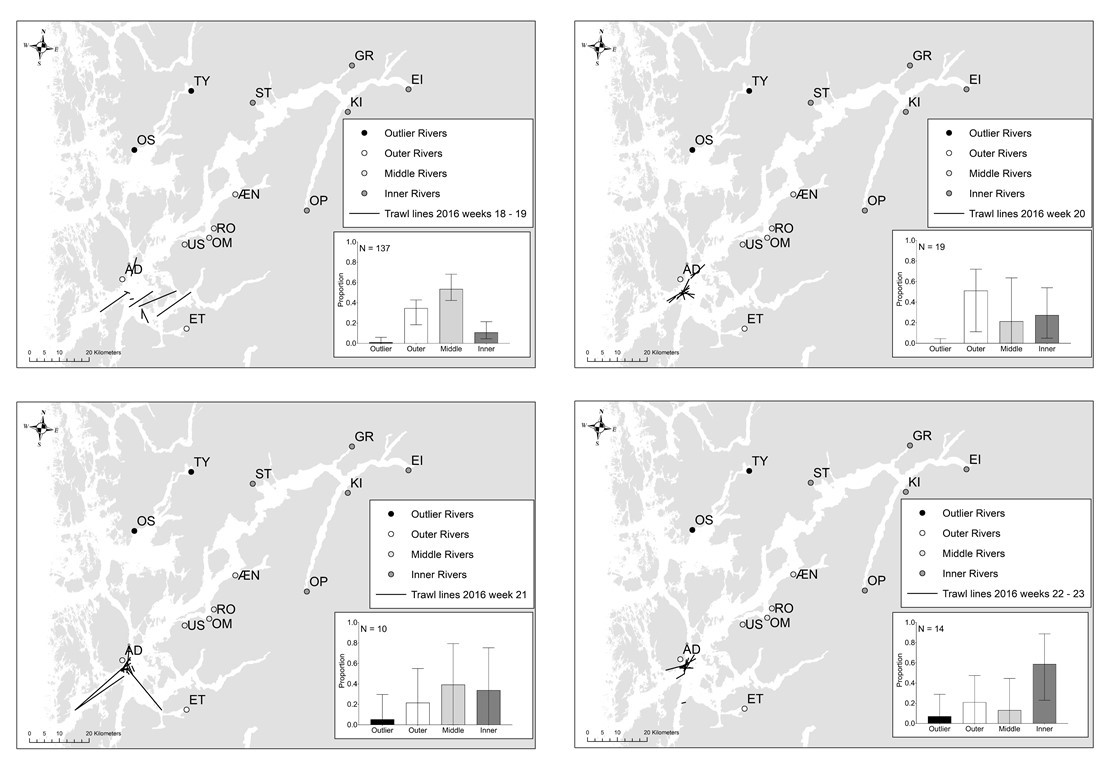


Figure S5: Weekly mixed stock analysis proportions of trawl fish caught in the 2016 trawl survey and trawl lines for each week.
